# Abnormal asymmetry correlates with abnormal enlargement in a patient with chronic moderate traumatic brain injury

**DOI:** 10.2217/cnc-2021-0006

**Published:** 2022-05-19

**Authors:** Justis Barcelona, David E Ross, John D Seabaugh, Jan M Seabaugh

**Affiliations:** 1 Department of Research, Virginia Institute of Neuropsychiatry, Midlothian, VA 23114, USA

**Keywords:** asymmetry, MRI, traumatic brain injury, volumetry

## Abstract

**Aim:**

Recent studies found patients with chronic, mild or moderate traumatic brain injury had more regions of enlargement than atrophy. There is little research discussing brain volume enlargement, asymmetry and TBI.

**Materials & methods:**

In this report, we describe a 40-year-old man who suffered a left cerebral hemorrhage resulting in a moderate TBI, suggesting greater forces on the left side of his brain. NeuroQuant^®^ brain volumetric analyses of his MRI obtained 1.7 years post injury showed left cerebral white matter atrophy but right gray matter abnormal enlargement. Abnormal asymmetry of multiple regions (R >L) was confirmed by NeuroGage^®^ asymmetry analyses.

**Discussion:**

The findings suggested that abnormal brain volume enlargement was due to hyperactivity and hypertrophy of less-injured brain regions as a compensatory response to more-injured regions.

Recent reports found that patients with chronic mild or moderate TBI had atrophy of cerebral white matter and a few other regions, but they had even more regions of abnormal enlargement, including cortical gray matter regions and subcortical regions [1–5].

Furthermore, decades of research have shown that patients with traumatic brain injury (TBI) have abnormal asymmetry of brain regions [6–8]. However, most of this research was based on patients with severe TBI. Much less is know about abnormal asymmetry (or its relationship with abnormal enlargement) in patients suffering from chronic effects of mild or moderate TBI [9,10].

The relatively new findings of abnormal enlargement raise the question: what is the mechanism behind the enlargement? Previously we proposed two hypotheses: chronic inflammation causes edema; and chronic dysfunction causes compensatory hypertrophy. For additional comments, see the Supplementary Material.

## Aims & hypotheses of the current report

This case report describes a patient with day-of-injury left-sided cerebral intraparenchymal bleeding, suggesting greater traumatic forces to his left than right cerebral hemisphere. Later brain volumetric analyses showed multiple regions of abnormal enlargement, consistent with the pattern commonly seen in patients with mild or moderate TBI. Asymmetry analyses were done in order to explore the possible relationship between the day-of-injury findings and later volumetric findings. In contrast with this patient, most patients with chronic mild or moderate TBI do not have intraparenchymal bleeding, and if they do have intraparenchymal bleeding, often it is not clearly unilateral. Therefore, these particular circumstances allowed for exploring hypotheses about asymmetry and their relationship to abnormally small or large volume.

The more forceful injury to the left cerebral hemisphere predicted that he would have more cerebral white matter atrophy on the left than right. The neuroinflammation hypothesis predicted that he also would have had more cortical gray matter enlargement on the left than right due to greater injury on the left. In contrast, right-sided cortical gray matter enlargement would have been more consistent with the less direct effect of compensatory hypertrophy.

## Brain imaging methods

NeuroQuant^®^ (NQ) 3.0 software was used to measure MRI brain volume and NeuroGage^®^ (NG) 3.0 software was used to measure brain volume asymmetry. For a description of these methods see [11] and the Supplementary Material.

The patient’s case reported herein exemplifies important aspects of using the latest NQ and NG methods to help understand the effects of brain injury on a single patient’s brain volume. To our knowledge, prior to the current report, there has been no peer-reviewed published case report using NQ 3.0 or NG 3.0.

The patient provided written informed consent for his imaging and clinical data to be published.

## Patient’s history & imaging

### Pre-accident history 

At the time of injury, patient RT was a 40-year-old man who, prior to the accident, had no neurological or psychiatric disorders.

### Date of incident

In July 2016, the patient was driving a 2011 trash collection truck, making a left turn. A tractor-trailer travelling at around 60 mph ran a red light and hit the left front side of the patient’s vehicle. He suffered a large scalp laceration, a right ear laceration/avulsion, a left clavicle fracture, fractures of several thoracic vertebra and a spinal cord injury.

**Figure 1.**
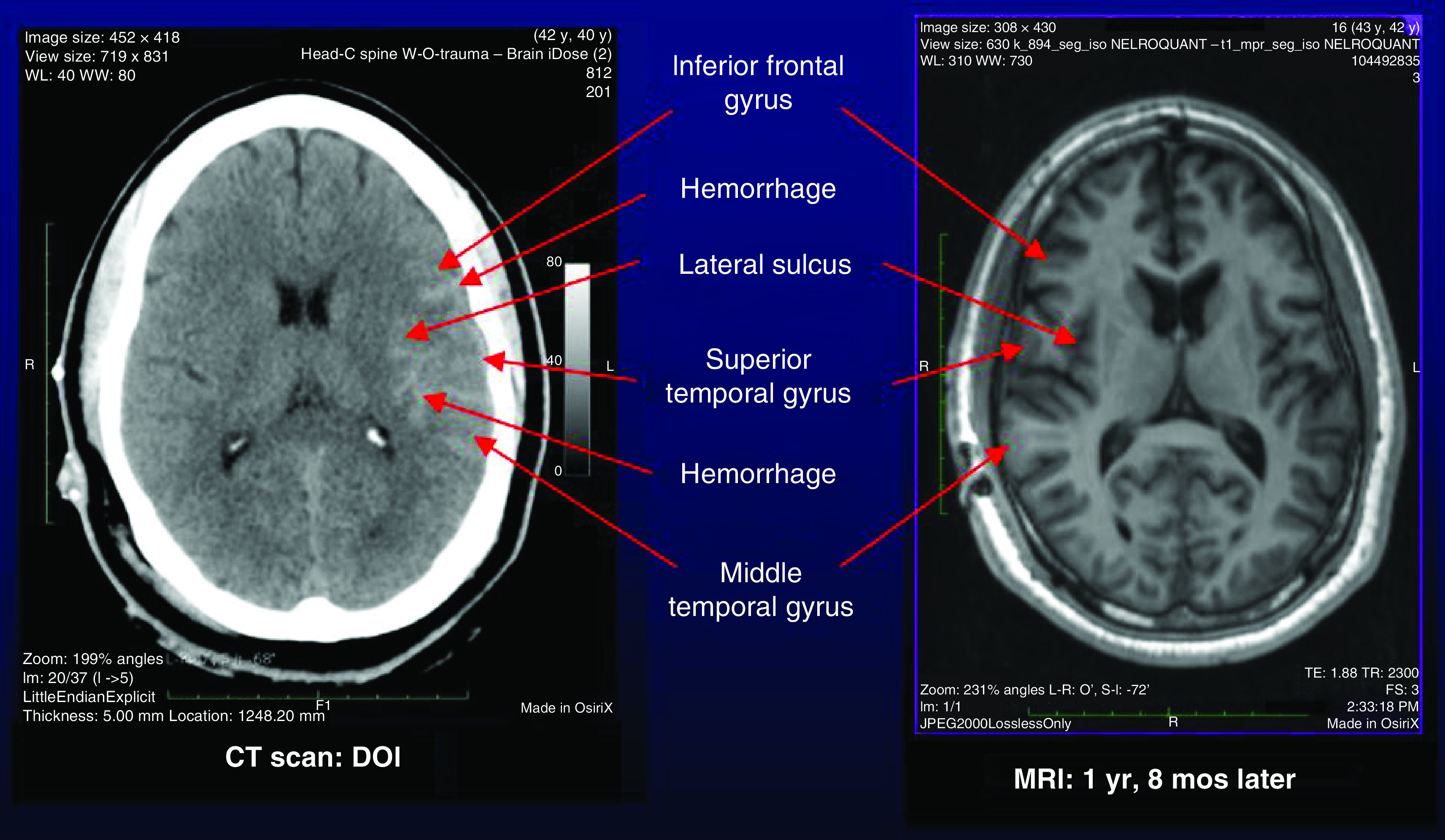
Patient RT had left cerebral hemorrhage on the day-of-injury CT scan, indicating greater forces to the left side of his brain. On follow-up MRI 1 year and 8 months later, he appeared to have normal brain structure. However, NeuroQuant^®^ and NeuroGage^®^ volume analyses showed left-sided cerebral atrophy, abnormal enlargement of many right-sided brain regions and abnormal asymmetry of many brain regions with R >L. These findings suggested that the right cerebral regions became enlarged due to compensatory hypertrophy.

Immediately after impact, he was unconscious for less than 30 min. He had no memory of the collision. The post-traumatic amnesia and altered consciousness persisted for several days. The emergency medical squad transported him to the hospital, where his Glasgow Coma Scale score was 10 (= avg of 8–12). CT scan on the date of injury showed left cerebral subarachnoid and intraparenchymal hemorrhage (Figure 1). Based on these data, he satisfied the diagnostic criteria for traumatic brain injury, and the severity of his traumatic brain injury was moderate [12,13].

Over the following months, he had extensive treatment and rehabilitation. Nevertheless, at the time of our initial evaluation of him in February 2018, he had multiple persistent neuropsychiatric symptoms typical of patients suffering from chronic effects of traumatic brain injury, including distractibility, impaired short-term memory, impaired verbal fluency, generalized anxiety, irritability, insomnia, fatigue, visuospatial impairment, photosensitivity, hyperacusis and chronic pain from multiple musculoskeletal injuries. These symptoms were debilitating to his work and social life. A comprehensive treatment and rehabilitation program was recommended (for additional comments, see the Supplementary Material).

### Patient RT: Follow-up MRI: NQ & NG analyses

Routine visual inspection of the MRI of the brain obtained 1.7 years post injury showed apparent resolution of the anatomic abnormalities (Figure 1). However, NeuroQuant 3.0 Triage Brain Atrophy analysis showed widespread volume abnormalities consistent with the group findings from our previous studies [5], including left cerebral white matter atrophy, abnormal enlargement of bilateral cerebellar white matter and right thalamus, and abnormal enlargement of multiple cortical gray matter regions (Table 1). There were 12 regions abnormally enlarged on the right side, versus only four regions enlarged on the left side, suggesting a possible overall pattern of abnormal asymmetry (R >L). NeuroGage asymmetry analyses confirmed this pattern, with ten regions showing abnormal asymmetry (R >L) versus only three showing abnormal asymmetry (L >R) (Table 2).

**Table 1.** NeuroQuant® 2.3 triage brain atrophy report.

	Percentiles		
	Left	Right	Total
**Total volumes**			
Cerebral white matter	3^†^	39	15
Cortical gray matter	93	92	93
Ventricles	87	80	85
– Subcortical structures:			
• Cerebellar white matter	99^‡^	99^‡^	99^‡^
• Cerebellar gray matter	92	91	92
• Brainstem	–	–	67
• Thalamus	29	96^‡^	76
• Ventral dencephalon	94	92	94
– Basal ganglia:			
• Putamen	18	63	37
• Caudate	58	94	83
• Nucleus accumbens	5	25	11
• Pallidum	32	80	56
– Cingulate:	90	67	81
• Anterior cingulate	67	9	24
• Posterior cingulate	99^‡^	99^‡^	99^‡^
• Isthmus cingulate	38	74	55
**Cortical brain regions**			
Frontal lobes:	69	55	62
• Superior frontal	29	24	25
• Middle frontal	61	7	25
• Inferior frontal	58	97^‡^	87
• Lateral orbitofrontal	55	79	68
• Medial orbitofrontal	77	26	44
• Paracentral	90	95	93
• Primary motor	90	75	85
Parietal lobes:	92	96^‡^	95
• Primary sensory	22	63	40
• Medial parietal	99^‡^	98^‡^	99^‡^
• Superior parietal	79	71	77
• Inferior parietal	96^‡^	92	97^‡^
• Supramarginal	74	97^‡^	90
Occipital lobes:	83	81	84
• Medial occipital	95	99^‡^	98^‡^
• Lateral occipital	56	37	45
Temporal lobes:	95	98^‡^	98^‡^
• Transverse temporal + superior temporal	88	95	94
• Posterior superior temporal sulcus	76	94	91
• Middle temporal	78	26	53
• Inferior temporal	90	45	76
• Fusiform	95	99^‡^	99^‡^
• Parahippocampal	78	90	86
• Entorhinal cortex	78	94	91
• Temporal pole	75	99^‡^	97^‡^
• Amygdala	4†	29	10
• Hippocampus	73	99^‡^	97^‡^

NeuroQuant® 2.3 Triage Brain Atrophy report for patient RT showed left cerebral white matter atrophy, and multiple regions of abnormal enlargement, with more regions of right-sided enlargement than left.

Intracranial volume: 1460.45 cm^3^.

Intracranial volume percentile: 2.

† Abnormally small parenchymal volume was defined by NeuroQuant as <5th normative percentile.

‡ Abnormally large volume was defined as >95th normative percentile.

**Table 2.** NeuroGage 2.3 Brain Volumetric Asymmetry report for patient RT showed multiple regions of abnormal asymmetry.

Brain region	LH		RH		Asymmetry index	Normative percentile
	Volume (cc)	ICV (%)	Volume (cc)	ICV (%)		
**NeuroGage^®^ asymmetry analysis for patient RT** ^†^						
Whole brain parenchyma	577.17	39.52%	602.77	41.27%	-4.34%	**0.34%** ^‡^
Forebrain parenchyma	489.60	33.52%	514.69	35.24%	-5.00%	**0.04%** ^‡^
Cerebral white matter	200.84	13.75%	219.47	15.03%	-8.86%	**0.00%** ^‡^
Total cortical gray matter	265.11	18.15%	268.88	18.41%	-1.41%	31.50%
Subcortical + infratentorial regions	111.22	7.62%	114.43	7.83%	-2.84%	15.57%
3rd ventricle	0.55	0.04%	1.28	0.09%	-79.76%	**2.13%** ^‡^
Lateral ventricle	10.39	0.71%	8.42	0.58%	20.98%	72.98%
Inferior lateral ventricle	0.20	0.01%	0.04	0.00%	130.29%	**99.46%** ^‡^
Cerebellum	77.49	5.31%	76.16	5.21%	1.74%	61.37%
Cerebellar white matter	20.66	1.41%	20.81	1.42%	-0.74%	24.27%
Cerebellar gray matter	56.84	3.89%	55.35	3.79%	2.66%	83.85%
Brainstem	10.09	0.69%	11.92	0.82%	-16.70%	30.51%
Thalamus	6.41	0.44%	7.36	0.50%	-13.80%	**0.05%** ^‡^
Ventral diencephalon	4.05	0.28%	3.82	0.26%	5.72%	46.80%
Hippocampus	3.70	0.25%	4.45	0.30%	-18.53%	**0.51%** ^‡^
Amygdala	1.34	0.09%	1.40	0.10%	-4.75%	12.15%
**Basal Ganglia**						
Putamen	4.34	0.30%	4.74	0.32%	-8.64%	**1.47%** ^‡^
Caudate	2.52	0.17%	3.12	0.21%	-21.04%	**1.90%** ^‡^
Nucleus accumbens	0.48	0.03%	0.52	0.04%	-8.14%	6.98%
Pallidum	0.80	0.05%	0.94	0.06%	-15.44%	13.74%
**Frontal Lobe**						
Precentral	13.69	0.94%	12.95	0.89%	5.50%	60.94%
Premotor	5.39	0.37%	4.50	0.31%	17.87%	78.16%
Superior frontal	27.45	1.88%	27.59	1.89%	-0.51%	26.20%
Anterior middle frontal	7.66	0.52%	7.04	0.48%	8.33%	**95.96%** ^‡^
Lateral orbito frontal	10.29	0.70%	9.70	0.66%	5.91%	34.44%
Pars orbitalis	4.39	0.30%	6.22	0.43%	-34.46%	11.82%
Primary motor	5.64	0.39%	5.01	0.34%	11.87%	65.27%
**Parietal lobe**						
Inferior parietal	13.87	0.95%	17.21	1.18%	-21.49%	56.93%
Superior parietal	12.66	0.87%	12.98	0.89%	-2.56%	59.40%
Medial parietal	13.60	0.93%	10.27	0.70%	27.86%	85.53%
Supramarginal	11.20	0.77%	9.89	0.68%	12.36%	17.48%
Primary sensory	9.62	0.66%	10.81	0.74%	-11.67%	**2.92%** ^‡^
**Occipital lobe**						
Medial occipital	18.83	1.29%	18.80	1.29%	0.16%	25.42%
Lateral occipital	16.80	1.15%	13.21	0.90%	23.96%	78.01%
**Temporal lobe**						
Fusiform	10.61	0.73%	14.43	0.99%	-30.57%	8.42%
Anterior medial temporal	3.15	0.22%	3.33	0.23%	-5.74%	22.59%
Posterior medial temporal	2.44	0.17%	2.76	0.19%	-12.08%	23.85%
Temporal pole	4.38	0.30%	5.23	0.36%	-17.72%	**4.09%** ^‡^
Transverse + superior temporal	19.45	1.33%	19.81	1.36%	-1.80%	14.32%
Posterior superior temporal sulcus	0.05	0.00%	0.30	0.02%	-147.67%	11.35%
Middle temporal	14.22	0.97%	13.57	0.93%	4.73%	80.16%
Inferior temporal	11.72	0.80%	10.14	0.69%	14.45%	**96.16%** ^‡^
**Limbic lobe**						
Caudal + rostral ant cingulate	1.80	0.12%	2.69	0.18%	-39.71%	44.50%
Isthmus + post cingulate	4.14	0.28%	3.93	0.27%	5.08%	30.70%

† He had many regions of abnormal asymmetry characterized by left side smaller than expected based on the mean volume of the left and right sides (L<R, or equivalently, R>L), findings which were consistent with cerebral white matter atrophy and abnormal enlargement of multiple right-sided regions found in the NeuroQuant^®^ Triage Brain Atrophy analysis.

‡ Abnormal asymmetry (L<R) was defined by NeuroGage as <5th normative percentile and abnormal asymmetry (R<L) was defined as >95th normative percentile.

Patient RT had many regions of abnormal asymmetry characterized by left side smaller than expected based on the mean volume of the left and right sides (L <R, or equivalently, R >L), findings which were consistent with cerebral white matter atrophy and abnormal enlargement of multiple right-sided regions found in the NeuroQuant® Triage Brain Atrophy analysis. Abnormal asymmetry (L <R) was defined by NeuroGage as <5th normative percentile (bold), and abnormal asymmetry (R <L) was defined as >95th normative percentile (bold).

ICV: Intracranial volume; LH: Left hemisphere; RH: Right hemisphere.

## Discussion

Patient RT’s findings suggested several causative mechanisms. The date-of-injury intraparenchymal cerebral hemorrhage on the left side but not the right made it likely that he had greater forces to his left cerebrum than right. These forces probably caused greater brain damage on the left, resulting in cerebral white matter atrophy on the left but not the right. This conclusion was supported by the following findings: his follow-up MRI and related NeuroQuant analysis showed atrophy of the left but not right cerebral white matter; and the related NeuroGage asymmetry analysis showed abnormal asymmetry of the cerebral white matter (L<R).

However, most of the left-sided cortical gray matter regions directly affected by the contusion did not have abnormally small volume at the time of follow-up. Instead, they mostly had normal volume, suggesting that they were less injured than the adjacent white matter or that they had healed since injury. But interestingly, many of the right-sided regions were abnormally large and had abnormal asymmetry (R >L), probably due to the injury, since he had no pre-injury diagnoses that would explain those abnormal findings.

We hypothesize that the left-sided injury caused cerebral white matter injury, rendering adjacent cortical gray matter less functional, resulting in compensatory hypertrophy of their contralateral counterpart brain regions.

Similarly, we hypothesize that cerebellar white matter becomes enlarged due to its compensating for injured or dysfunctional cerebral regions. The cerebellum coordinates movement and – to a lesser extent – thought and emotion; and therefore, it plays an important support role for most cerebral functions [14,15].

The data for this case example did not support the neuroinflammation hypothesis, which predicted more enlargement on the left side of the brain due to greater injury than the right.

There were several correlations between patient RT’s abnormal volume findings and his clinical symptoms, based on the known function of the respective brain regions. Atrophy and abnormal asymmetry of the cerebral white matter correlated with bradyphrenia [16–18] and executive dysfunction [18]. Abnormal asymmetry of the thalamus correlated with impaired sleep and wakefulness [19]. Abnormally large volume of the posterior cingulate gyri correlated with impaired mood [20]. Abnormal volume of the precuneus correlated with impaired visuospatial skills [21]. Abnormal volume of the medial occipital cortex (composed of the lingual gyrus and cuneus) correlated with impaired visual system; the medial occipital cortex is necessary for both basic and higher level visual processing [22]. Abnormally large volume of the transverse temporal + superior temporal cortical region correlated with hyperacusis (transverse temporal gyri) [23]. Abnormal asymmetry and enlargement of the right temporal pole correlated with impaired mood [24] and impaired emotional empathy/irritability [25]. Abnormally large volume and asymmetry of the hippocampus correlated with impaired short-term memory [26].

Unfortunately, but as is typical for cases like this, there was no pre-accident brain imaging available. Therefore, we could not definitively prove that patient RT’s brain volume changed abnormally from before to after injury. However, we believe that our conclusions have heuristic value and are justified at least in at least a preliminary way based on the following considerations: he had no pre-accident neuropsychiatric disorders, making it unlikely that he had much if any abnormal volume; he had day-of-injury brain scanning that showed acute intracerebral bleeding, which is well-known to be associated with later atrophy; he had later atrophy (supported by the volumetric analyses) as expected; he had later abnormal asymmetry which supported the idea (although did not prove) that abnormal volume changes on one side of the brain caused the asymmetry.

## Conclusion

In summary, these results showed that day-of-injury left-sided bleeding led to chronic left-sided cerebral white matter atrophy but right-sided cortical gray matter enlargement. These findings supported the hypothesis of compensatory hypertrophy, that is, that the injury caused cerebral white matter atrophy on a given side of the brain, leading to hypertrophy of contralateral brain regions in an effort to compensate for the more injured ipsilateral regions.

**Summary points**

- Although decades of research have found extensive brain atrophy in patients with severe traumatic brain injury (TBI), more recent studies have found substantial brain volume enlargement in patients with chronic mild or moderate TBI.
- This case report describes a 40-year-old man with brain imaging findings that suggested a hypothesis explaining why brain regions become enlarged in many patients with chronic mild or moderate TBI.
- Patient RT had a day-of-injury left cerebral hemorrhage resulting in a moderate TBI, suggesting greater forces on the left side of his brain.
- Volume analyses conducted 1.8 years after injury showed left cerebral white matter atrophy and asymmetry (L <R), also suggesting greater forces on the left side of his brain.
- In contrast to the findings regarding cerebral white matter, volume analyses also conducted in the chronic phase showed multiple regions of right-sided cortical gray matter enlargement and asymmetry (R >L).
- The findings suggested that abnormal brain volume enlargement was due to hyperactivity and hypertrophy of less-injured brain regions as a compensatory response to more-injured regions.
- Similarly, we hypothesize that cerebellar white matter became enlarged due to its compensating for injured or dysfunctional cerebral regions.
- The data for this case example did not support the neuroinflammation hypothesis, which predicted more enlargement on the left side of the brain due to greater injury than the right.
