# Supplementary material for: Abnormal asymmetry correlates with abnormal enlargement in a patient with chronic moderate traumatic brain injury
Source: Concussion. 2022 May 19;7(1):CNC96. doi: 10.2217/cnc-2021-0006 (PMC9219597; doi:10.2217/cnc-2021-0006)
Supplement: Supplementary file 1 [file cnc-07-96-s1.docx]

# Supplemental comments on: Introduction

## 2 hypotheses for enlargement

### Inflamm hypothesis

In support of the inflammation hypothesis, studies in animals and humans have found that moderate to severe traumatic brain injury causes both acute and chronic neuroinflammation.([Donkin and Vink 2010](#_ENREF_2), [Bigler and Maxwell 2012](#_ENREF_1), [Smith, Gentleman et al. 2012](#_ENREF_22), [Finnie 2013](#_ENREF_3), [Johnson, Stewart et al. 2013](#_ENREF_5), [Kou and VandeVord 2014](#_ENREF_6), [Lozano, Gonzales-Portillo et al. 2015](#_ENREF_7), [Taib, Leconte et al. 2017](#_ENREF_23)) And neuroinflammation potentially can occur for years after TBI.([Johnson, Stewart et al. 2013](#_ENREF_5)) Therefore, it seems possible that chronic neuroinflammation and edema could cause the abnormally large brain volume in patients with chronic mild or moderate TBI.

### Compens hypertrophy hypothesis

Regarding the possibility of compensatory hypertrophy, enlargement could occur in the regions injured, or in secondary regions connected to the injured regions. It is well-known that when brain regions perform more tasks, they enlarge in volume; classic studies of this phenomenon include the keyboard player study([Gaser and Schlaug 2003](#_ENREF_4)) and the London taxi driver study.([Maguire, Gadian et al. 2000](#_ENREF_8))

## Aims and hypotheses of the current report

This case report describes a patient with day-of-injury left-sided cerebral intraparenchymal bleeding, suggesting greater traumatic forces to his left than right cerebral hemisphere. Later brain volumetric analyses showed multiple regions of abnormal enlargement, consistent with the pattern commonly seen in patients with mild or moderate TBI. Asymmetry analyses were done in order to explore the possible relationship between the day-of-injury findings and later volumetric findings.

The more forceful injury to the left cerebral hemisphere predicted that he would have more cerebral white matter atrophy on the left than right. The neuroinflammation hypothesis predicted that he also would have had more cortical gray matter enlargement on the left than right due to greater injury on the left. In contrast, right-sided cortical gray matter enlargement would have been more consistent with the less direct effect of compensatory hypertrophy.

# Supplemental comments on: Brain imaging methods:

## Intro

Previously we have described our use of NeuroQuant (software versions 1.x and 2.x) and NeuroGage (software versions 1.0 and 2.0) for analyzing MRI brain volume ([Ross, Ochs et al. 2012](#_ENREF_14), [Ross, Castelvecchi et al. 2013](#_ENREF_11), [Ross, Graham et al. 2013](#_ENREF_12), [Ross, Ochs et al. 2013](#_ENREF_15), [Ross, Ochs et al. 2014](#_ENREF_17), [Ochs, Ross et al. 2015](#_ENREF_9), [Ross, Ochs et al. 2015](#_ENREF_13), [Ross, Ochs et al. 2016](#_ENREF_18), [Ross, Ochs et al. 2018](#_ENREF_16), [Ross, Seabaugh et al. 2018](#_ENREF_19), [Ross, Seabaugh et al. 2020](#_ENREF_21), [Ross, Seabaugh et al. 2021](#_ENREF_10), [Ross, Seabaugh et al. in press; 2022](#_ENREF_20)). These studies include descriptions of our methods for asymmetry analyses ([Ross, Ochs et al. 2015](#_ENREF_13), [Ross, Seabaugh et al. 2018](#_ENREF_19)). Herein, previously described methods will be described briefly, and more recent methods will be described in more detail.

## NQ

NeuroQuant is FDA-cleared software for measuring MRI brain volume (<https://www.cortechs.ai/products/neuroquant>). In 2019, version 3.0 was released (followed by version 3.1 in May 2021) (for sample NeuroQuant^®^ 3.0 reports, see online supplemental files: <https://www.dropbox.com/sh/jcha6dtjzhxwax1/AABrvd89ltboAOAPRpTJIHqUa?dl=0>). NeuroQuant identifies brain regions and compares the patient’s volumes to age- and sex-matched normal comparison data. Compared to earlier versions, NeuroQuant^®^ 3.0 has several improvements, including the following: 1) More accurate segmentation algorithms, that is, algorithms for identifying brain regions (for sample NeuroQuant^®^ 3.0 segmented brain images, see online supplemental file: <https://www.dropbox.com/sh/jcha6dtjzhxwax1/AABrvd89ltboAOAPRpTJIHqUa?dl=0>. 2) Identification of more brain regions (over 130 regions). 3) A larger normal control database: about 4000 normal controls ranging in age from 3 to 100 years; personal communication with Weidong Luo, Ph.D., Principal Scientist, CorTechs Labs Inc., March 23, 2020).

## NG

NeuroGage is software that is based on NeuroQuant and that extends the utility of NeuroQuant in several ways, including providing asymmetry analyses. NeuroGage asymmetry analyses are designed to assess whether the difference between left- and right-sided counterpart brain regions are greater than would be expected to occur normally. NeuroGage and NeuroQuant measure asymmetry with the asymmetry index, defined in the traditional way, as follows:

1) Calculate the difference between left and right-sided volumes of the counterpart structures = left volume – right volume = L-R.

2) Calculate the mean of the left and right-sided volumes = (left volume + right volume)/2 = (L+R)/2.

3) Calculate the asymmetry index = difference/mean = (L-R)/[(L+R/2)].

Note that the denominator (= mean of L and R volumes) can be thought of as the best estimate of pre-injury volume for the left or right-sided brain region. Thus, the asymmetry index is measure of how much the left and right volumes differ relative to the estimated pre-injury volume of each structure.

The latest version of NeuroGage^®^ is 3.0 is based on NeuroQuant^®^ 3.0 (for a sample NeuroGage^®^ report, see online supplemental file: <https://www.dropbox.com/sh/jcha6dtjzhxwax1/AABrvd89ltboAOAPRpTJIHqUa?dl=0>. Compared to NeuroGage^®^ 2.0, version 3.0 was improved in several ways, including a larger number of brain regions (52 regions) for asymmetry analyses. The patient’s data were compared to the NeuroGage normal control data, with asymmetry (L<R) defined as an asymmetry index < 5^th^ normative percentile, and asymmetry (R<L) defined as an asymmetry index > 95^th^ normative percentile.

# Supplemental comments on: Clinical history

On the day of the collision (in late July 2016) at the hospital, Mr. Thompson was found to have a GCS which was waxing and waning between 8 to 12. He underwent RSI (rapid sequence induction) intubation in the trauma bay. His scalp laceration required immediate surgical fixation. He also underwent repair of his right ear laceration/avulsion. In the surgical intensive care unit, he was noted to be flaccid in his lower extremities. A MRI of this thoracic spine showed epidural hematoma and posttraumatic disk extrusion at T3, associated with severe canal stenosis. He emergently underwent bilateral T2, T3, T4, and T5 posterior lateral fusion, and T3 and T4 laminectomy for decompression.

The day after the collision, he was able to be extubated. He was confused and disoriented due to the traumatic brain injury. The neurologist’s diagnoses included encephalopathy related to traumatic brain injury.

He was transferred to surgical step-down unit. He had periods of low-grade fever and leukocytosis of 12,000. Chest x-ray showed atelectasis. He was started on ceftriaxone. He was seen by PT, OT, and Speech Pathology. He was in need of ongoing spinal cord rehab therapy.

11 days after the collision, in early August 2018 , he underwent left clavicular surgical correction (ORIF).

Two days later, he was transferred from the acute care hospital to a rehabilitation specialty hospital. While there, evaluation by speech language pathology showed cognitive impairment due to the brain injury. Also he was evaluated by physical therapy and occupational therapy.

There was initial concern for his traumatic brain injury where agitation was an issue. A brain injury physician recommended starting quetiapine. The agitation was controlled, his mood became stable, and the quetiapine was discontinued prior to discharge.

Head CT scan was repeated, and it showed a resolving subarachnoid hemorrhage. His agitation had reduced significantly.

Both postoperative pain and neuropathic pain were initially treated with oxycodone as needed and gabapentin, which was increased to 300 mg tid. Over time, he no longer required the oxycodone. He had a trigger point injection for scapular dyskinesis, with reported resolution of pain in this area toward the end of his stay.

He was started on baclofen 5 mg tid for spasticity, which was increased to 20 mg qid.

In late September 2016, he was discharged from the rehabilitation hospital to home. Discharge medications included gabapentin for pain, baclofen for spasticity, acetaminophen for pain, and trazodone for insomnia.

With physical therapy, a Bioness leg apparatus and botulinum toxin injections, Mr. Thompson was able to walk with a cane.

In November 2017, Mr. Thompson received a baclofen pump (implanted under the skin of his right lower abdomen) to treat spasticity. He continued taking oral baclofen while the pump dose was being titrated.

In mid-October 2016, he was followed up by a neurosurgeon. He had regained complete strength back in his legs. However, his walking was somewhat hampered by spasticity in his left leg. “Overall, this was amazing improvement.”

In early November 2016, he was evaluated by a neurologist for leg and back pain. He ambulated with a walker. He felt he had some muscle spasticity in the legs that was controlled with baclofen. However, his main concern was feeling of band-like tightness or odd sensation in the legs. He stated it was not painful, but was bothersome. He stated when he sat or lay down, he had a pressure or band-like sensation involving the entirety of both legs. Gabapentin helped reduce these sensations. The neurologist opined that the band-like sensations in his legs was due to the spinal cord dysfunction.

In early January 2017, he was evaluated by a neuropsychologist. He exhibited abnormally variable performance on the neuropsychological tests administered, with scores ranging from 2nd to 87th percentile. Performance at the 10th percentile or less were noted on measures of fund of knowledge, auditory attention, problem solving, and visuoconstruction. Vocationally, he appeared to be at risk for failure. He performed at ≤10th percentile on measures of visual reasoning, auditory attention, and visuoconstruction.

In May 2017, his physiatrist noted that he underwent botulinum toxin injection into the left quadriceps in March. He reported 75% improvement. It was still helping. He used a single-point cane with quadripod extension. He was using the Bioness L300 Plus. He was doing outpatient physical therapy 2x/week and NeuroFit 2x/week. He was making significant improvement. He denied pain.

In September 2017, his physiatrist noted that patient RT was still dealing with increased spasticity in both legs. Severity was 8/10. He described a tightness across his waist and in both legs. It affected his walking. He was doing NeuroFit twice weekly and physical therapy three times a week and was doing his own stretching at home. He increased his baclofen to 40 mg qid and gabapentin 800 mg qid but this combination was too strong. He was now taking gabapentin 400 mg alternating with 800 mg qid. He decreased baclofen back to 40 mg tid. His wife felt the gabapentin was too strong. He was give a injection of botulinum toxin in his left quadricep. The plan included referral for intrathecal baclofen administration.

In late February 2018, we initially evalauted him. Our initial recommendations for treatment and rehabilitation for patient RT included the following:

- Consider Bose noise reduction headphones and Vibes ear plugs to reduce hyperacusis.
- Consider sunglasses, tinted lenses, and hat with brim (like baseball cap), even indoors, to reduce photosensitivity
- Check the following blood tests in order to assess for possible causes of neuropsychiatric symptoms:

| Comprehensive metabolic panel (14) (fasting) |
| --- |
| Complete blood count |
| Lipid panel (fasting) |
| Free T3 |
| Free T4 |
| Thyroid-stimulating hormone (TSH) |
| Vitamin B12 |
| Vitamin D |
| Ferritin |
| Testosterone, direct (for men) |

- 3.0 Tesla MRI of brain including the following:
- Qualitative (traditional) interpretation by radiologist
- Susceptibility-weighted imaging (SWI) sequence to look for signs of old bleeding
- Diffusion tensor imaging (DTI) sequence to examine white matter integrity
- Volumetric imaging sequence followed offline by brain volume analyses, including NeuroQuant^®^ and NeuroGage^®^
- Occupational therapy
- Cognitive rehabilitation with a speech/language therapist
- Individual or group psychotherapy and TBI coaching
- Cognitive desensitization therapy (for example, eye movement desensitization and reprocessing therapy or exposure therapy) for posttraumatic stress disorder with a trained therapist.
- Sleep consult and study
- Consult with physiatrist.
- Wheelchair access at his home, including ramp to enter and exit his house, ramp to allow him to move between different levels of his house, and chair lift for stairs.
- Driving evaluation by certified driving rehabilitation specialist (CDRS)
- Hyperbaric oxygen therapy for TBI
- Increase gabapentin from 400 mg qid as follows:
  - Day 1: 500 mg qid
  - Day 3: 600 mg qid
  - Day 5: 700 mg qid
  - Day 7: 800 mg qid according to clinical response.

Late March 2018 (1 month after initial evaluation): Gabapentin 800 mg qid led to no leg pain (but he still had leg spasticity), but sweating and hypersomnolence. Decreasing dose to 700 mg qid and then 600 mg qid led to no adverse effects but persistent benefit for leg pain, i.e. no leg pain.

The plan was to finish titrating and optimizing the dose of gabapentin. Then no sooner than 4 days later, add duloxetine to target pain, dysphoria and subthreshold PTSD.

In mid-April 2018, he reported that duloxetine 30 mg bid led to benefit of no leg spasticity and tolerable sweating. The plan was to try to taper and discontinue the gabapentin, to see if duloxetine by itself maintained the benefits.

In late May 2018, he reported that reducing the gabapentin led to worsening of leg pain, so he increased it back to 600 mg qid. He had increased insomnia for which a trial of doxylamine was recommended.

Follow up was recommended but he never returned to us for follow up.

References

Bigler, E. D. and W. L. Maxwell (2012). "Neuropathology of mild traumatic brain injury: relationship to neuroimaging findings." Brain Imaging Behav **6**(2): 108-136.

Donkin, J. J. and R. Vink (2010). "Mechanisms of cerebral edema in traumatic brain injury: therapeutic developments." Current Opinion in Neurology **23**: 293-299.

Finnie, J. W. (2013). "Neuroinflammation: beneficial and detrimental effects after traumatic brain injury. Inflammopharmacology." Inflammopharmacology **21**(4): 309-320.

Gaser, C. and G. Schlaug (2003). "Brain structures differ between musicians and non-musicians." J Neurosci **23**(27): 9240-9245.

Johnson, V. E., J. E. Stewart, F. D. Begbie, J. Q. Trojanowski, D. H. Smith and W. Stewart (2013). "Inflammation and white matter degeneration persist for years after a single traumatic brain injury." Brain **136**(Pt 1): 28-42.

Kou, Z. and P. J. VandeVord (2014). "Traumatic white matter injury and glial activation: from basic science to clinics." Glia **62**(11): 1831-1855.

Lozano, D., G. S. Gonzales-Portillo, S. Acosta, I. d. l. Pena, N. Tajiri and Y. Kaneko (2015). "Neuroinflammatory responses to traumatic brain injury: etiology, clinical consequences, and therapeutic opportunities." Dis Treat **2015**(11): 97-106.

Maguire, E. A., D. G. Gadian, I. S. Johnsrude, C. D. Good, J. Ashburner, R. S. Frackowiak and C. D. Frith (2000). "Navigation-related structural shange in the hippocampi of taxi drivers." Proceedings of the National Academy of Sciences of the United States of America **97**(8): 4398-4403.

Ochs, A. L., D. E. Ross, M. D. Zannoni, T. J. Abildskov and E. D. Bigler (2015). "Comparison of automated brain volume measures obtained with NeuroQuant and FreeSurfer." Journal of Neuroimaging **25**: 721-727.

Ross, D., J. Seabaugh, J. Seabaugh, J. Plumley, J. Ha, J. Burton, A. Vandervaart, R. Mischel, A. Blount, D. Seabaugh, K. Shepherd, J. Barcelona and A. Ochs (2021). "Patients with chronic mild or moderate traumatic brain injury have abnormal longitudinal brain volume enlargement more than atrophy." Journal of Concussion **5**: 1-21.

Ross, D. E., C. Castelvecchi and A. L. Ochs (2013). "Brain MRI volumetry in a single patient with mild traumatic brain injury." Brain Injury **27**: 634-636.

Ross, D. E., T. J. Graham and A. L. Ochs (2013). "Review of the evidence supporting the medical and forensic use of NeuroQuant® in patients with traumatic brain injury." Psychological Injury and the Law **6**: 75-80.

Ross, D. E., A. L. Ochs, M. E. DeSmit, J. M. Seabaugh and M. D. Havranek (2015). "Man vs. machine part 2: Comparison of radiologists’ interpretations and NeuroQuant® measures of brain asymmetry and progressive atrophy in patients with traumatic brain injury." Journal of Neuropsychiatry and Clinical Neurosciences **27**: 147-152.

Ross, D. E., A. L. Ochs, J. M. Seabaugh, M. F. DeMark, C. R. Shrader, J. H. Marwitz and M. D. Havranek (2012). "Progressive brain atrophy in patients with chronic neuropsychiatric symptoms after mild traumatic brain injury: A preliminary study." Brain Injury **26**: 1500-1509.

Ross, D. E., A. L. Ochs, J. M. Seabaugh and C. R. Shrader (2013). "Man vs. machine: Comparison of radiologists’ interpretations and NeuroQuant® volumetric analyses of brain MRIs in patients with traumatic brain injury." Journal of Neuropsychiatry and Clinical Neurosciences **25**: 1-8.

Ross, D. E., A. L. Ochs, D. F. Tate, U. Tokac, J. Seabaugh, T. J. Abildskov, E. D. Bigler and For_the_Alzheimer’s_Disease_Neuroimaging_Initiative (2018). "High correlations between MRI brain volume measurements based on NeuroQuant® and FreeSurfer." Psychiatry Research Neuroimaging **278**: 69-76.

Ross, D. E., A. L. Ochs, M. D. Zannoni and J. M. Seabaugh (2014). "Back to the future: Estimating pre-injury brain volume in patients with traumatic brain injury." NeuroImage **102**: 565-578.

Ross, D. E., A. L. Ochs, M. D. Zannoni and J. M. Seabaugh (2016). "Corrigendum to “Back to the Future: Estimating Pre-Injury Brain Volume in Patients with Traumatic Brain Injury” [NeuroImage 102 (Part 2) (15 November 2014) 565–578]." NeuroImage **127**: 510-511.

Ross, D. E., J. Seabaugh, L. Cooper and J. M. Seabaugh (2018). "NeuroQuant® and NeuroGage® reveal effects of traumatic brain injury on brain volume." Brain Injury **32**: 1437-1441.

Ross, D. E., J. Seabaugh, J. M. Seabaugh, J. Barcelona, D. Seabaugh, K. Wright, L. Norwind, Z. King, T. J. Graham, J. Baker and T. Lewis (in press; 2022). "Updated review of the evidence supporting the medical and legal use of NeuroQuant® and NeuroGage® in patients with traumatic brain injury." Frontiers in Human Neuroscience.

Ross, D. E., J. D. Seabaugh, J. M. Seabaugh, C. Alvarez, L. P. Ellis, C. Powell, C. Hall, C. Reese, L. Cooper and A. L. Ochs (2020). "Patients with chronic mild or moderate traumatic brain injury have abnormal brain enlargement." Brain Injury **34**: 11-19.

Smith, C., S. M. Gentleman, P. D. Leclercq, L. S. Murray, W. S. T. Griffin and D. L. Graham (2012). "The neuroinflammatory response in humans after traumatic brain injury." Neuropathology and Applied Neurobiology **39**(6): 654-666.

Taib, T., C. Leconte, J. V. Steenwinkel, A. H. Cho, B. Palmier, E. Torsello, R. L. Kuen, S. Onyeomah, K. Ecomard, C. Benedetto, B. Coqueran, A. C. Novak, E. Deou, M. Plotkine, P. Gressens, C. M. Leroux and V. C. Besson (2017). "Neuroinflammation, myelin and behavior: Temporal patterns following mild traumatic brain injury in mice." PLoS One.
